# HDR syndrome with a novel mutation in *GATA3* mimicking a congenital X-linked stapes gusher: a case report

**DOI:** 10.1186/s12881-017-0484-6

**Published:** 2017-10-26

**Authors:** Aram Yang, Jinsup Kim, Chang-Seok Ki, Sung Hwa Hong, Sung Yoon Cho, Dong-Kyu Jin

**Affiliations:** 1 Department of Pediatrics, Samsung Medical Center, Sungkyunkwan University School of Medicine, 81 Irwon-ro, Gangnam-gu, Seoul, 06351 Korea; 2 Department of Laboratory Medicine and Genetics, Samsung Medical Center, Sungkyunkwan University School of Medicine, Seoul, Korea; 3 Department of Otorhinolaryngology-Head and Neck Surgery, Samsung Changwon Hospital, Sungkyunkwan University School of Medicine, Seoul, Korea

**Keywords:** HDR syndrome, *GATA3* gene, Hearing loss, Sensorineural, Hypoparathyroidism, Renal anomalies, DFNX2, Case report

## Abstract

**Background:**

Hypoparathyroidism, sensorineural hearing loss, and renal disease (HDR) syndrome, also known as Barakat syndrome, is a rare genetic disorder with high phenotypic heterogeneity caused by haploinsufficiency of the *GATA3* gene on chromosome 10p14-p15. For these reasons, the diagnosis of HDR syndrome is challenging and requires a high index of suspicion as well as genetic analysis.

**Case presentation:**

A 14-month-old boy, with sensorineural hearing loss in both ears, showed typical radiological features of X-linked stapes gusher on preoperative temporal bone computed tomography (CT) for cochlear implantations. Then after his discharge from hospital, he suffered a hypocalcemic seizure and we discovered a renal cyst during investigation of hypocalcemia. He was finally diagnosed with HDR syndrome by clinical findings, which were confirmed by molecular genetic testing. Direct sequencing of the *GATA3* gene showed a heterozygous 2-bp deletion (c.1201_1202delAT), which is predicted to cause a frameshift of the reading frame (p.Met401Valfs*106).

**Conclusions:**

To our knowledge, this is the first case of HDR syndrome with a novel de novo variant mimicking a congenital X-linked stapes gusher syndrome. Novel mutations and the diversity of clinical manifestations expand the genotypic and phenotypic spectrum of HDR syndrome.

Diagnosis of HDR syndrome is still challenging, but clinicians should consider it in their differential diagnosis for children with a wide range of clinical manifestations including hypocalcemia induced seizures and deafness. We hope that this case will contribute to further understanding and studies of HDR-associated GATA3 mutations.

**Electronic supplementary material:**

The online version of this article (10.1186/s12881-017-0484-6) contains supplementary material, which is available to authorized users.

## Background

HDR syndrome (OMIM 146255) was first reported in 1977 as a rare autosomal dominant disorder characterized by hypoparathyroidism, SNHL, and renal disease [1–3].

After Van Esch et al. [4] revealed that the haploinsufficiency of *GATA3* located on chromosome 10p14-p15 causes human HDR syndrome through deletion-mapping studies in two HDR patients, there have been many studies of the *GATA3* gene related to HDR syndrome [5].

*GATA3* is one of six members of a family of transcription factors that bind the consensus motif A/TGATAA/G and is expressed in the parathyroid, kidney, inner ear, thymus and central nervous system during embryonic development [4, 6]. In recent studies, a major role of *GATA3* includes otic morphogenesis in the signaling of prosensory genes and in the differentiation of cochlear neurosensory cells, but the whole process has not been fully elucidated [7–9]. Although the relationship between *GATA3* and inner ear development has been studied, a case of HDR syndrome with sensorineural deafness mimicking an X-linked stapes gusher has not previously been reported. X-linked deafness with stapes gusher, also referred as X-linked deafness-2 (DFNX2, OMIM 304400), is characterized by progressive conductive and sensorineural hearing loss and a pathognomonic temporal bone deformity that includes dilatation of the internal auditory canal (IAC) and a fistulous connection between the IAC and the cochlear basal turn. This results in a profuse flow of perilymph and cerebrospinal fluid (CSF) along with increased perilymphatic pressure after surgery to a congenitally fixed stapes footplate [10, 11]. Meanwhile, patients with HDR syndrome with severe SNHL requiring cochlear implantations due to congenital ear malformation have not been identified.

Here, we report the first case of HDR syndrome with a novel de novo mutation mimicking a congenital X-linked stapes gusher syndrome.

## Case presentation

A 14-month-old male with congenital sensorineural deafness was admitted to an ear, nose and throat (ENT) ward at Samsung Medical Center for cochlear implantation. The patient was born by vaginal delivery, without perinatal problems, at gestational age 34 + 0 weeks having a weight of 2.3 kg. He was the first-born child of non-consanguineous Korean parents with no known family history of hearing loss.

Hearing loss in both ears was detected at 3 months of age by brainstem evoked response audiometry (BERA) as part of an initial screening test. Additional serial audiological evaluation included repeated BERA that were not evocable at maximum stimulation levels, otoacoustic emissions (OAEs) that were absent bilaterally, auditory steady state response (ASSR) without hearing aids that revealed a profound hearing loss with residual hearing only at low frequencies. Thus, the diagnosis of SNHL was made by the age of 11 months. Vital signs upon admission found a blood pressure of 100/61 mmHg, heart rate of 131 beats/min, respiratory rate of 24 breaths/min, and body temperature of 36.6 °C. His height and weight were 76 cm (−0.7 SDS) and 9.5 kg (−1.3 SDS), respectively. Physical and neurological examinations were normal.

**Fig. 1:**
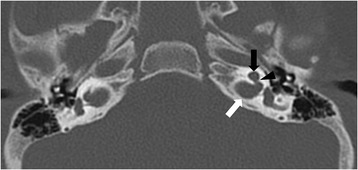
Computed tomography image of the temporal bone of a patient with HDR mimicking a congenital X-linked stapes gusher syndrome. Axial CT section of the temporal bones shows severe cochlear hypoplasia (*black arrow*), bulbous dilatation of the fundi of the IAC (white arrow) and a fistulous connection between the internal auditory canal and the cochlear basal turn with absence of the bony partitions (*black arrowhead*) on the left. These findings were seen on the right side (not shown) IAC; Internal Auditory Canal

Preoperative computed tomography (CT) and magnetic resonance imaging (MRI) of the temporal bones showed bulbous dilatation of the internal auditory canals, bilateral cochlear hypoplasia and a fistulous connection between the internal auditory canal and the cochlear basal turn suggestive of congenital X-linked stapes gusher syndrome (Fig. 1). We conducted gene analysis of *POU3F4,* which is responsible for X-linked stapes gusher syndrome; however, this mutation was not detected.

The initial laboratory studies during preoperative work up presented the following results: total calcium 7.0 mg/dL (reference range [RR], 8.4–10.2 mg/dL), phosphorus level: 7.5 mg/dL (RR, 2.5–4.5 mg/dL), ionized calcium 0.83 mmol/L (RR, 1.05–1.35 mmol/L), magnesium 2.2 mg/dL (RR, 1.9–2.5 mg/dL), serum alkaline phosphatase 204 U/L (RR, 180–380 IU/L). The serum intact-parathyroid hormone (PTH) level measured by immunoradiometric assay (IRMA) was 6.8 pg/mL (normal range: 11.2–62.0 pg/mL) and the level of 25(OH)-vitamin D was 21.90 ng/mL (RR, 30–100 ng/mL). The calcium-creatinine ratio in randomly collected urine samples was 0.13 (normal rage: < 0.3). On the fifth day of preoperative evaluation for cochlear implants, he was discharged with oral calcium supplements for asymptomatic hypocalcemia due to hypoparathyroidism.

Five days later, he was brought to the pediatric emergency room (ER) of our hospital following a brief generalized tonic-clonic seizure for a few seconds. At that time his serum calcium, ionized calcium and phosphorous levels were 6 mg/dL, 0.83 mmol/L, 8 mg/dL, each respectively. Electroencephalogram (EEG) and brain MRI at that time were unrevealing. He was treated with a single dose of 10% calcium gluconate solution intravenously. In addition, oral medication with calcium and vitamin D supplementation was started. Although the patient was not obviously dysmorphic, the presence of seizures due to secondary hypocalcemia, in association with hypoparathyroidism during early childhood, prompted fluorescence in situ hybridization (FISH) analysis for DiGeorge syndrome. His karyotype was normal. There were no abnormal findings detected by echocardiography and ophthalmologic examination.

Because he exhibited both SNHL and hypoparathyroidism, HDR syndrome was suspected.

To investigate suspected kidney problems, a kidney ultrasound (US) was performed, and the result revealed a 1.6 cm sized renal cyst in the left kidney. Finally, the combination of hypoparathyroidism, congenital deafness, and renal anomalies let us reach a diagnosis of HDR syndrome.

He was discharged with calcium supplements (75 mg/kg/day) and alfacalcidol (0.1 μg/kg), and exhibited normocalcemia (serum calcium 9.3 mg/dL) at the last examination. There have been no subsequent seizures and his milestones and development are normal. In addition, he showed advanced development of language skills following the cochlear implantation.

For identification of an underlying genetic cause for the observed symptoms, mutational analysis for the *POU3F4* and *GATA3* genes was performed. This study was approved by the institutional review board at Samsung Medical Center. After obtaining informed consent from both parents, genomic DNA was extracted from peripheral blood leukocytes of the patient and his parents using a Wizard genomic DNA purification kit (Promega, Madison, WI) according to the manufacturer’s instructions.

All coding exons and flanking intronic regions of the *POU3F4* and *GATA3* genes were amplified by polymerase chain reaction (PCR) using primers designed by the authors (available upon request). Cycle sequencing was performed with the BigDye Terminator Cycle Sequencing Ready Reaction kit (Applied Biosystems, Foster City, CA) on an ABI 3730 Genetic Analyzer (Applied Biosystems). The sequence chromatograms obtained were compared with the reference sequences (*POU3F4*, NM_000307.4; *GATA3*, NM_001002295.1). Identified variants were described following the recommendations of the Human Genome Variation Society (http://www.hgvs.org/mutnomen/).

**Fig. 2:**
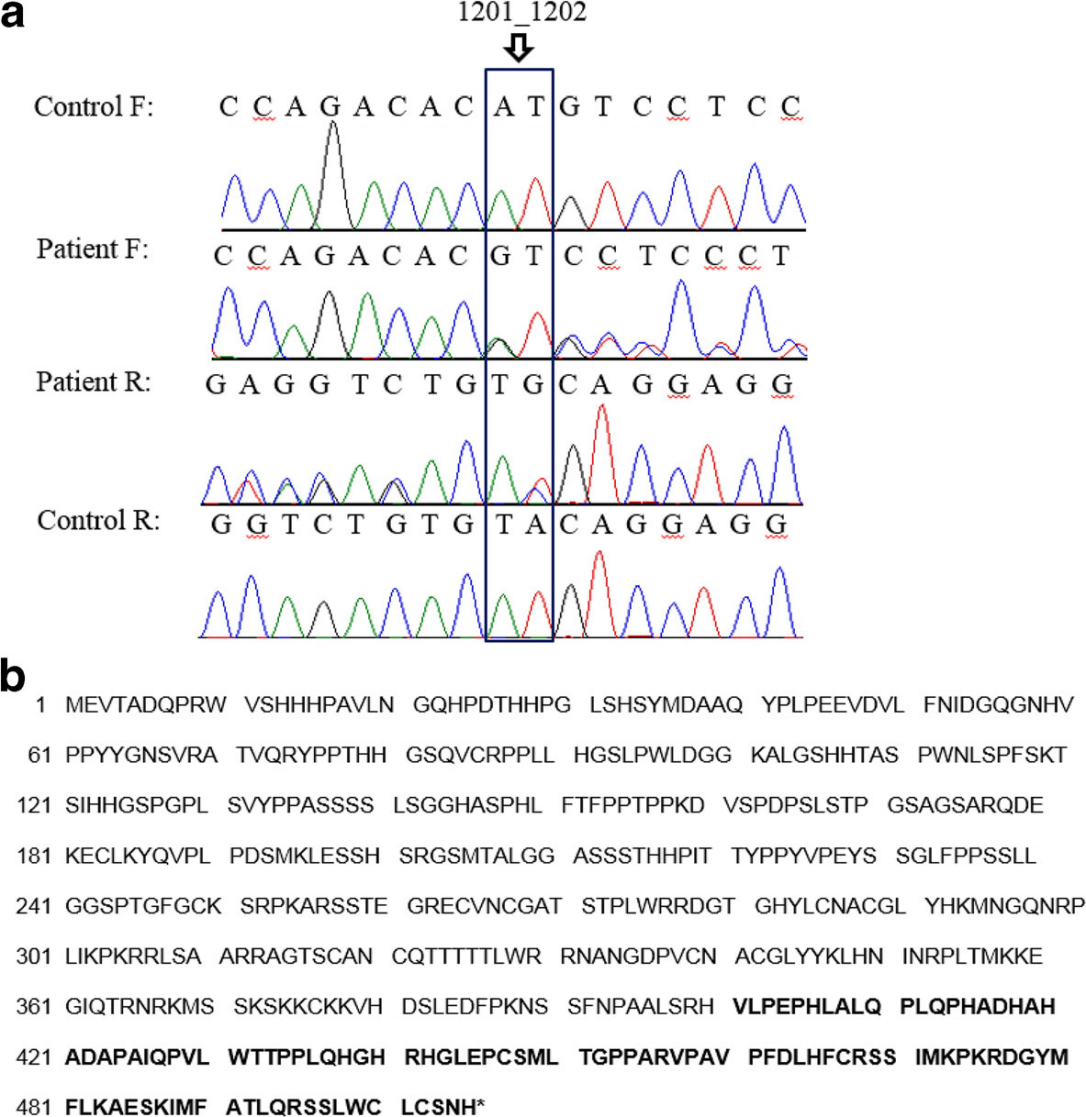
**a** Electropherogram of part of *GATA3* exon 6. Our case had a heterozygous 1201_1202del AT of the *GATA3* gene. Sequences of the control and the patient alleles are reported. A control reveals any carriers with the c. 1201_1202del AT. F and R indicate forward and reverse sequencings, respectively. **b** Wild-type sequence and predicted amino acid sequence resulting from frameshift mutation. Bold type indicates the new amino acid sequence

The patient was heterozygous for a 2-bp deletion at position 1201 to 1202 (c.1201_1202delAT) in exon 6 of the *GATA3* gene, which is predicted to cause a frameshift of the reading frame (p.Met401Valfs*106) (Fig. 2). Both parents did not have this variant; therefore, this variant is a de novo mutation (Additional file 1: Figure S2). No pathogenic variant was observed in the *POU3F4* gene. The CARE guidelines were followed in this study. The case report timeline is presented in Additional file 2: Figure S1.

## Discussion and Conclusions

We describe the case of HDR syndrome resulting from a novel de novo heterozygous mutation of GATA3 presented with hypocalcaemia, seizure, both SNHL with severe inner ear malformations mimicking DFNX2, and a simple renal cyst.

Over 90% of patients with HDR syndrome present with hypoparathyroidism, sensorineural deafness, and over 80% exhibit urinary tract and renal abnormalities [12].

Hypoparathyroidism manifests diverse presentations among HDR patients, ranging from asymptomatic hypocalcemia to paresthesia, muscular aching, and tetany, with low, normal, or even slightly elevated serum PTH levels [13]. Some patients with HDR syndrome may initially present with a hypocalcemia induced seizure like our patient.

The renal manifestations of HDR syndrome can include renal hypoplasia or aplasia, renal dysplasia, and vesico-ureteric reflux, nephrotic syndrome, a cystic kidney or chronic renal failure [12, 14, 15]. Prognosis of HDR syndrome depends on the nature and severity of any associated renal disease. Some patients may progress to chronic renal failure and require renal replacement therapy [1]. In addition, there has been a report about renal cyst progression in younger patients [16]. Thus, regular renal US is needed for our patient despite the diagnosis of a simple renal cyst.

SNHL is the most consistent feature of the HDR syndrome, which is usually bilateral (but may be asymmetric), and it ranges from mild to severe [17]. In recent studies, the *GATA3* mutation has been related to inner ear malformations [9, 18], however, there has been no previous report of HDR syndrome accompanied by severe congenital ear anomalies such as the X-linked stapes gusher syndrome.

X-linked deafness with stapes gusher is a rare anomaly associated with a mutation in *POU3F4* located on the X chromosome. It can be diagnosed based on profound SNHL, including a conductive hearing loss component in some cases, and typical imaging findings. In addition, like our patient, there has been sporadic reporting of de novo mutation of DFNX2 cases without any family history of deafness [19].

We initially suspected X-linked deafness with stapes gusher in view of the clinical history and the typical CT findings, including a bulbous dilatation of the fundi of the IAC canals, hypoplasia of the base of the cochleae and abnormal connection between the IAC and the basal turn of the cochlea, which were shown during preoperative assessment for the cochlear implants. During his cochlear implantation, which is known to be a viable option for a patient with SNHL suspected with X-linked stapes gusher, perilymphatic gusher did not occur.

Although mutation of *POU3F4* was not detected in this patient, we cannot completely rule out the diagnostic possibility of DFNX2. Since there could be a significant degree of undetected deletion or duplication of the *POU3F4* gene and there may be involvement of unidentified genes in *Xq21*.*1* that can cause DFNX2. Therefore, work-up of hypocalcemia and any renal anomaly might be recommended for a patient with congenital deafness and severe inner ear deformity.

The molecular analysis of our patient revealed a heterozygous deletion of AT nucleotides in exon 6 (c.1201_1202delAT) of the *GATA3* gene, which is predicted to cause a frameshift at the 401st codon for the methionine and the new reading frame ending in a stop at position 106 (p.Met401Valfs*106). It is a novel de novo mutation in exon 6 of the *GATA3* gene associated with HDR syndrome.

A previously reported Japanese patient with a similar frameshift mutation in exon 6 of the *GATA3* gene (c.1200_1201delCA; p.H400fsX506) characteristically showed insulin dependent diabetes with a more severe renal anomaly compared to our patient [20]. In addition to these phenotypic differences, the variant in our patient was different from most other reported *GATA3* frameshift mutations. Instead of resulting in a loss of the two zinc finger domains ZnF1 and ZnF2 of *GATA3*, p.Met401Valfs*106 is expected to result in extended protein length while preserving ZnF1 and ZnF2 because they occur downstream [2]. Although Ali et al. (2007) [21] reported a frameshift insertion in codon 407 which predicts the occurrence of 98 missense residues leading to extended amino acids, the patient did not manifest stapes gusher as our patient did. The wide phenotypic spectrum of *GATA3* mutations that cause HDR syndrome (missense, nonsense, frameshifting indels, whole gene deletions) support the critical role of functional GATA3 protein dosage — all mutations result in the loss of functional *GATA3*, whether total or partial loss of function.

To date, more than 50 *GATA3* mutations have been reported in patients with HDR (http://www.hgmd.cf.ac.uk/ac/index.php). Several *GATA3* mutation types are related to HDR syndrome, including deletion, base substitution, missense, frameshift, insertion and splice mutation [1]. The *GATA3* gene consists of six exons and encodes a 444-amino acid transcriptional factor containing two transactivating domains (TA1 and TA2) at the N-terminus and two zinc fingers domains at the C-terminus (ZnF1 and ZnF2) (see Additional file 3: Figure S3) [22]. Functional analysis of the mutated *GATA3* gene revealed that ZnF2 is essential for DNA binding, whereas the ZnF1 stabilizes binding to DNA and interacts with other proteins [2].

In recent studies, the importance of *GATA3* mutation has been recognized due to the multiple functions of *GATA3* involved in regulatory T-cell function, multi-organ differentiation, and tumorigenesis as a potential predictor for the outcome of breast cancer [23, 24]. It is especially noteworthy that frameshift insertions leading to C-terminal extensions are the most common GATA3 mutations found in breast cancer [25, 26]. Based on these newly detected diverse functions of *GATA3*, further investigation of the possible role of a novel gene, *GATA3*, will be worthwhile to determine if there is a genotype-phenotype relationship in HDR syndrome.

Despite variations in the phenotypic presentation of HDR syndrome, early specific diagnosis of it is important. First, understanding the molecular basis of HDR syndrome through genetic analysis and providing appropriate genetic counseling is significant for patients with HDR syndrome and their families to know the exact cause of their disease and to prepare for any subsequent pregnancy. Second, close monitoring of hypocalcemia and elective medical treatment can prevent serious hypocalcemic seizures following a brain damage.

Conducting renal US, audiometry, developing a detailed family history, genetic analysis of *GATA3,* and a work-up for hypocalcemia is highly recommended in any patient with hypocalcemia induced seizures associated with deafness. This information is needed so that physicians can identify this very rare genetic disorder of HDR syndrome.

In this study, we present the first case of HDR syndrome with a novel de novo variant mimicking a congenital X-linked stapes gusher syndrome. Our case highlights the wide clinical spectrum and severity of HDR syndrome, which poses a great challenge to arrive at a molecular diagnosis. Nevertheless, early recognition of the HDR syndrome is crucial, not only for genetic counselling but also to offer a more precise prognosis and elective therapy for prevention of hypocalcemic seizures. Further study should investigate the phenotypic spectrum of mutations in the GATA3 gene.

## Additional files

Additional file 1: Figure S2. Sequencing results of the patient and his parents with reverse primer. As the *HDR* mutation observed in the affected child was not present in any of the parents, they represent de novo mutations. (TIFF 1424 kb)

Additional file 2: Figure S1. Case report timeline. Presented according to CARE guidelines. (TIFF 2843 kb)

Additional file 3: Figure S3. Schematic genomic structure of the *GATA3* gene: *GATA3* contains 6 exons and white boxes indicate a non-coding region, and black boxes indicate conding region. The arrow indicates the mutation identified in the patient. (TIFF 120 kb)

## Abbreviations

DFNX2: X-linked deafness-2
IAC: Internal auditory canal
SNHL: Sensorineural hearing loss

## References

[1] Muroya K, Hasegawa T, Ito Y, Nagai T, Isotani H, Iwata Y, Yamamoto K, Fujimoto S, Seishu S, Fukushima Y (2001). GATA3 abnormalities and the phenotypic spectrum of HDR syndrome. J Med Genet.

[2] Nesbit MA, Bowl MR, Harding B, Ali A, Ayala A, Crowe C, Dobbie A, Hampson G, Holdaway I, Levine MA (2004). Characterization of GATA3 mutations in the hypoparathyroidism, deafness, and renal dysplasia (HDR) syndrome. J Biol Chem.

[3] Hasegawa T, Hasegawa Y, Aso T, Koto S, Nagai T, Tsuchiya Y, Kim KC, Ohashi H, Wakui K, Fukushima Y (1997). HDR syndrome (hypoparathyroidism, sensorineural deafness, renal dysplasia) associated with del(10)(p13). Am J Med Genet.

[4] Van Esch H, Groenen P, Nesbit MA, Schuffenhauer S, Lichtner P, Vanderlinden G, Harding B, Beetz R, Bilous RW, Holdaway I (2000). GATA3 haplo-insufficiency causes human HDR syndrome. Nature.

[5] Bernardini L, Sinibaldi L, Capalbo A, Bottillo I, Mancuso B, Torres B, Novelli A, Digilio MC, Dallapiccola B (2009). HDR (Hypoparathyroidism, deafness, renal dysplasia) syndrome associated to GATA3 gene duplication. Clin Genet.

[6] Mino Y, Kuwahara T, Mannami T, Shioji K, Ono K, Iwai N (2005). Identification of a novel insertion mutation in GATA3 with HDR syndrome. Clin Exp Nephrol.

[7] Luo XJ, Deng M, Xie X, Huang L, Wang H, Jiang L, Liang G, Hu F, Tieu R, Chen R (2013). GATA3 controls the specification of prosensory domain and neuronal survival in the mouse cochlea. Hum Mol Genet.

[8] Duncan JS, Fritzsch B (2013). Continued expression of GATA3 is necessary for cochlear neurosensory development. PLoS One.

[9] Haugas M, Lillevali K, Salminen M (2012). Defects in sensory organ morphogenesis and generation of cochlear hair cells in Gata3-deficient mouse embryos. Hear Res.

[10] Song MH, Lee HK, Choi JY, Kim S, Bok J, Kim UK (2010). Clinical evaluation of DFN3 patients with deletions in the POU3F4 locus and detection of carrier female using MLPA. Clin Genet.

[11] de Kok YJ, van der Maarel SM, Bitner-Glindzicz M, Huber I, Monaco AP, Malcolm S, Pembrey ME, Ropers HH, Cremers FP (1995). Association between X-linked mixed deafness and mutations in the POU domain gene POU3F4. Science.

[12] Kato Y, Wada N, Numata A, Kakizaki H (2007). Case of hypoparathyroidism, deafness and renal dysplasia (HDR) syndrome associated with nephrocalcinosis and distal renal tubular acidosis. Int J Urol.

[13] Hernandez AM, Villamar M, Rosello L, Moreno-Pelayo MA, Moreno F, Del Castillo I (2007). Novel mutation in the gene encoding the GATA3 transcription factor in a Spanish familial case of hypoparathyroidism, deafness, and renal dysplasia (HDR) syndrome with female genital tract malformations. Am J Med Genet A.

[14] Ranjbar-Omrani G, Zamiri N, Sabayan B, Mohammadzadeh A (2008). Concomitant hypoparathyroidism, sensorineural deafness, and renal agenesis: a case of Barakat syndrome. Arch Iran Med.

[15] Sepahi MA, Baraty B, Shooshtary FK (2010). HDR syndrome (Hypoparathyroidism, Sensorineural deafness and renal disease) accompanied by Hirschsprung disease. Iran J Pediatr.

[16] Terada N, Ichioka K, Matsuta Y, Okubo K, Yoshimura K, Arai Y (2002). The natural history of simple renal cysts. J Urol.

[17] Maleki N, Bashardoust B, Iranparvar Alamdari M, Tavosi Z (2013). Seizure, deafness, and renal failure: a case of Barakat syndrome. Case Rep Nephrol.

[18] Chien WW, Leiding JW, Hsu AP, Zalewski C, King K, Holland SM, Brewer C (2014). Auditory and vestibular phenotypes associated with GATA3 mutation. Otol neurotol.

[19] Moteki H, Shearer AE, Izumi S, Kubota Y, Azaiez H, Booth KT, Sloan CM, Kolbe DL, Smith RJ, Usami S (2015). De novo mutation in X-linked hearing loss-associated POU3F4 in a sporadic case of congenital hearing loss. Ann Otol Rhinol Laryngol.

[20] Muroya K, Mochizuki T, Fukami M, Iso M, Fujita K, Itakura M, Ogata T (2010). Diabetes mellitus in a Japanese girl with HDR syndrome and GATA3 mutation. Endocr J.

[21] Ali A, Christie PT, Grigorieva IV, Harding B, Van Esch H, Ahmed SF, Bitner-Glindzicz M, Blind E, Bloch C, Christin P (2007). Functional characterization of GATA3 mutations causing the hypoparathyroidism-deafness-renal (HDR) dysplasia syndrome: insight into mechanisms of DNA binding by the GATA3 transcription factor. Hum Mol Genet.

[22] Yang Z, Gu L, Romeo PH, Bories D, Motohashi H, Yamamoto M, Engel JD (1994). Human GATA-3 trans-activation, DNA-binding, and nuclear localization activities are organized into distinct structural domains. Mol Cell Biol.

[23] Wang Y, MA S, Wan YY (2011). An essential role of the transcription factor GATA-3 for the function of regulatory T cells. Immunity.

[24] Du F, Yuan P, Wang T, Zhao J, Zhao Z, Luo Y, Xu B (2015). The significance and therapeutic potential of GATA3 expression and mutation in breast cancer: a systematic review. Med Res Rev.

[25] Gaynor KU, Grigorieva IV, Nesbit MA, Cranston T, Gomes T, Gortner L, Thakker RV (2009). A missense GATA3 mutation, Thr272Ile, causes the hypoparathyroidism, deafness, and renal dysplasia syndrome. J Clin Endocrinol Metab.

[26] Mair B, Konopka T, Kerzendorfer C, Sleiman K, Salic S, Serra V, Muellner MK, Theodorou V, Nijman SM (2016). Gain- and loss-of-function mutations in the breast cancer gene GATA3 result in differential drug sensitivity. PLoS Genet.

