# Supplementary figures and images for: HDR syndrome with a novel mutation in GATA3 mimicking a congenital X-linked stapes gusher: a case report
Source: BMC Med Genet. 2017 Oct 26;18:121. doi: 10.1186/s12881-017-0484-6 (PMC5659003; doi:10.1186/s12881-017-0484-6)

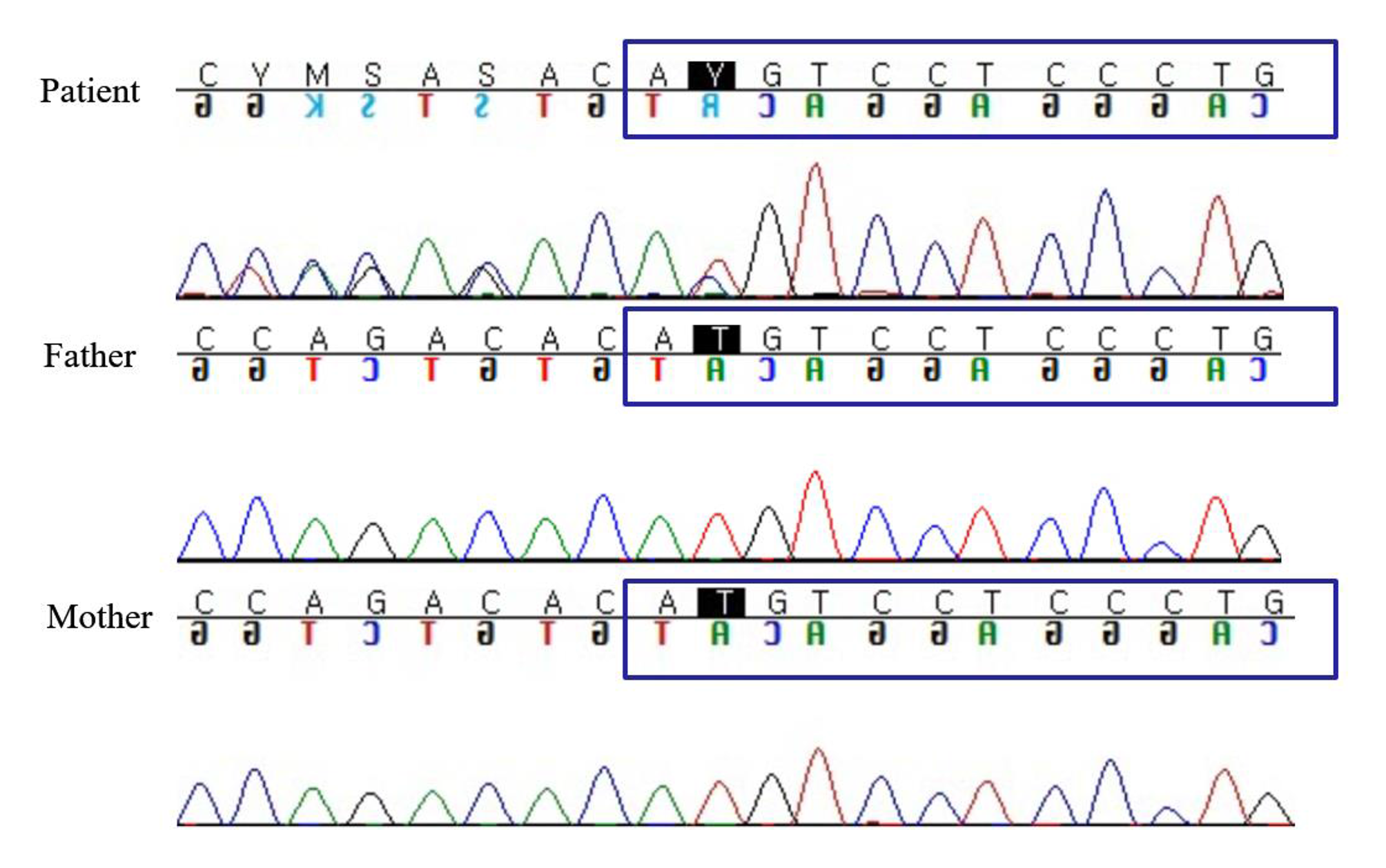

Supplement: Supplementary file 1 — Sequencing results of the patient and his parents with reverse primer. As the HDR mutation observed in the affected child was not present in any of the parents, they represent de novo mutations. (TIFF 1424 kb) [file 12881_2017_484_MOESM1_ESM.tif]

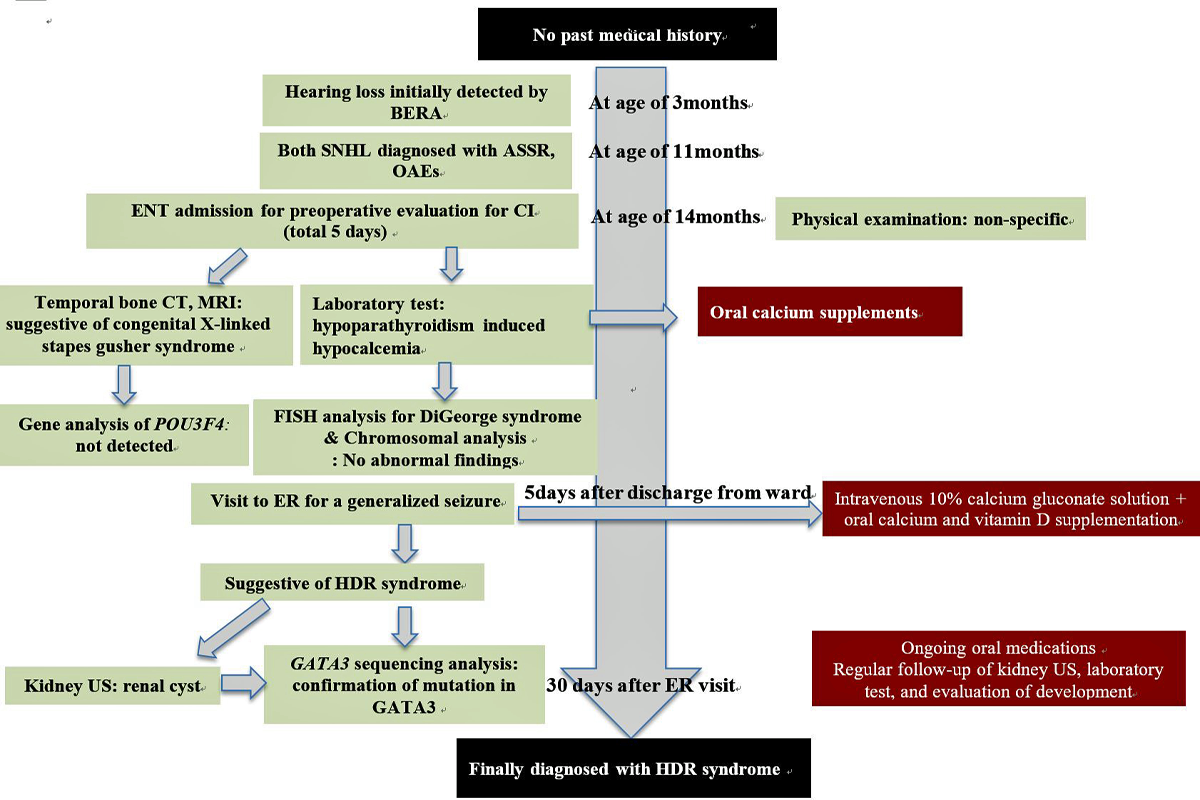

Supplement: Supplementary file 2 — Case report timeline. Presented according to CARE guidelines. (TIFF 2843 kb) [file 12881_2017_484_MOESM2_ESM.tif]

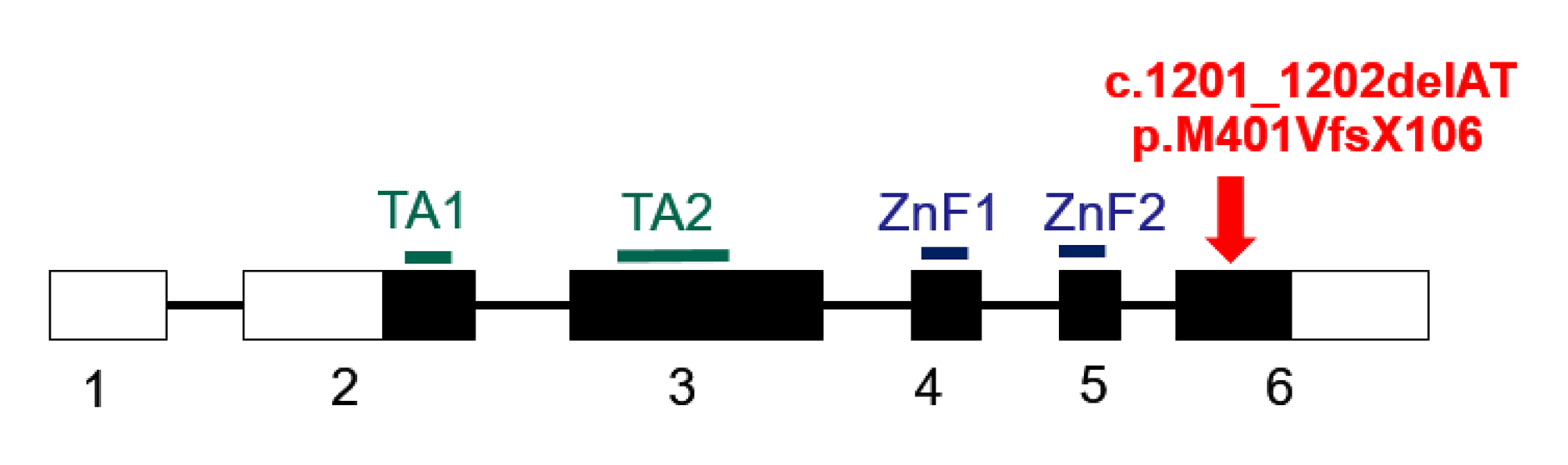

Supplement: Supplementary file 3 — Schematic genomic structure of the GATA3 gene: GATA3 contains 6 exons and white boxes indicate a non-coding region, and black boxes indicate conding region. The arrow indicates the mutation identified in the patient. (TIFF 120 kb) [file 12881_2017_484_MOESM3_ESM.tif]
